# 

**Published:** 1894-01

**Authors:** 


### CONTENTS:

ORIGINAL ARTICLES: PAGE

New Remedies in Veterinary Medicine. By Fred. W. Turner, Ph.G., D.V.S ........
Osteo-Porosis. By G. J. Bowker, V.S........
Chronic Bright's Disease, with Renal Calculi, in a Bitch. By Andrew Darling, M.D., D.V.S ........ 10
Tuberculin in Practice. By Cooper Curtice........ 11
The Dog in Human Society: His Great Faults and the Remedy. By Alexander Hadden, M.D........ 17
Ruptured Bladder. By Leon N. Reefer, V.M.D........ 27
Influenza. By Dr. W. B. E. Miller........ 28
Veterinary Colleges ; a Correction. By James R. Waugh, V.S........ 34
Iatrol as an Antiseptic. By. Dr. D. Eugene Black........ 35
A Study in Horse Shoeing By Williamson Bryden, V.S........ 36
Treatment of Staggers. By M. Parazols........ 40
Osteo-Porosis, *not* Millet Disease. By W. H. Mattsen, V.M.D........ 41
Quittor. By Williamson Bryden, V.S........ 43
Tubercular Meningitis. By W. H. Mattsen, V.M.D........ 47

EDITORIAL:

Current Literature for Veterinarians........ 48

SOCIETY PROCEEDINGS :

Veterinary Medical Association of New Jersey........ 55
Keystone Veterinary Medical Association........ 56
Montreal Veterinary Medical Association........ 57
California State Veterinary Medical Association........ 58
Ontario Veterinary Association, Annual Meeting........ 65

Errata........ 66
Annual Meeting New York State Veterinary Medical Society........ xiv

